# Behavior at Air/Water Interface and Oxidative Stability of Vegetable Oils Analyzed Through Langmuir Monolayer Technique

**DOI:** 10.3390/molecules30010170

**Published:** 2025-01-04

**Authors:** Wiktoria Kamińska, Katarzyna Rzyska-Szczupak, Anna Przybylska-Balcerek, Kinga Stuper-Szablewska, Anna Dembska, Grażyna Neunert

**Affiliations:** 1 Department of Physics and Biophysics, Faculty of Food Science and Nutrition, Poznan University of Life Sciences, Wojska Polskiego 38/42, 60-637 Poznan, Poland; 2 Department of Chemistry, Faculty of Forestry and Wood Technology, Poznan University of Life Sciences, 60-628 Poznan, Poland; katarzyna.rzyska@up.poznan.pl (K.R.-S.); anna.przybylska@up.poznan.pl (A.P.-B.); kinga.stuper@up.poznan.pl (K.S.-S.); 3 Department of Bioanalytical Chemistry, Faculty of Chemistry, Adam Mickiewicz University, Uniwersytetu Poznanskiego 8, 61-614 Poznan, Poland

**Keywords:** fatty acids, vegetable oils, Langmuir monolayer, oxidation stability

## Abstract

This study aimed to evaluate the oxidative stability and surface properties of cold-pressed vegetable oils using the Langmuir monolayer technique. Six oils—milk thistle, evening primrose, flaxseed, camelina sativa, black cumin, and pumpkin seed—were analyzed to investigate their molecular organization and behavior at the air/water interface, particularly after undergoing oxidation. The results showed that oils rich in polyunsaturated fatty acids (PUFAs), such as flaxseed and evening primrose oils, formed monolayers with larger molecular areas and lower stability, which led to faster oxidative degradation, especially under thermal conditions. In contrast, pumpkin seed oil, with a higher content of saturated fatty acids (SFAs), formed more condensed and stable monolayers, enhancing its resistance to oxidation. Black cumin oil, with a balanced profile of SFAs and monounsaturated fatty acids (MUFAs), demonstrated similar stability. The Langmuir technique facilitated a detailed analysis of monolayer phase transitions: PUFA-rich oils transitioned more readily to less stable phases, while SFA-rich oils maintained durable, condensed structures. These findings underscore the utility of this method for assessing the oxidative stability of vegetable oils and highlight key parameters—such as surface pressure, molecular area, and elasticity modulus—that can support the optimization of oil storage and quality in the food industry and related sectors.

## 1. Introduction

Oils and fats are essential components of the human diet worldwide. In recent years, dietary changes aligned with healthy eating principles have led to a reduction in the intake of saturated fatty acids (SFAs) and an increase in the consumption of monounsaturated (MUFAs) and polyunsaturated fatty acids (PUFAs) [1,2,3]. This trend has driven a growing demand for alternative vegetable oils low in SFAs and high in MUFAs and PUFAs. Vegetable oils are complex mixtures of fatty acids, phospholipids, natural dyes, and antioxidants [4]. They play a key role in the diet by providing energy, essential fatty acids, vitamins (A, D, E, and K), and other bioactive compounds, supporting proper bodily functions [5,6]. Vegetable oils are particularly important for physiological functions like growth and development, as they contain structural components of cell membranes and act as carriers for fat-soluble compounds. Moreover, fats and their lipid components enhance the taste and texture of food, contributing to the characteristics of many food products [7,8].

Oxidation is a significant chemical process that negatively impacts the quality and stability of food products, especially fats and oils. Oxidative degradation, driven by exposure to oxygen, light, and heat, leads to rancidity and the formation of harmful compounds. The oxidative stability of oils depends on their fatty acid composition and the presence of antioxidants, which vary by oil type. The higher the degree of unsaturation in the fatty acids, and the lower the content of saturated fatty acids, the faster the oxidation process occurs [9,10]. Various methods and tests are used to assess oxidative stability, including the accelerated storage test, which simulates oxidative aging by exposing samples to elevated temperatures (Schaal oven test). This test evaluates the stability and shelf life of food products, particularly fats and oils, by accelerating chemical reactions that lead to degradation [11,12,13]. Among the chemical analyses used to evaluate the oxidative stability of oils and fats, the specific absorbance coefficient of oil (*K*_232_, *K*_268_) has shown a good correlation with the content of unsaturated fatty acids [14]. Other methods used, such as peroxide value (PV), do not always accurately reflect the observed stability of the studied oils [15], as they may be influenced by the presence of different pigments in the oil. Additionally, titration methods demonstrate low reproducibility due to variability in handling empirical titration assays [16].

The Langmuir monolayer method serves as a two-dimensional model for studying molecular structures, providing insights into the interactions, packing, and orientation of molecules at phase boundaries. It involves forming a thin monolayer at the gas/liquid interface by compressing surfactants. During the measurement, changes in surface pressure (π) are recorded as a function of the surface area available per molecule (π-A isotherm) [17,18,19]. The characterization and analysis of thin surface layers are often complemented by other techniques. Among experimental methods used to study adsorbed layers at phase boundaries are surface rheology techniques. Surface rheological properties, which depend on interactions between molecules and relaxation kinetics, allow for studying layer structure and the mechanisms governing these processes. Surface deformation can be broken down into shear and dilatational components, enabling the differentiation of their properties [19,20,21].

The application of Langmuir monolayers combined with advanced techniques like oscillating barriers spans various fields, from food science to material engineering. According to our knowledge, previous research utilizing Langmuir monolayers for food-related compounds has focused on characterizing model monolayers of fatty acids, lipids, and proteins [22,23]. However, studies on more complex systems such as vegetable oils remain limited. Only a few studies have applied the Langmuir method to analyze the behavior and properties of oil mixtures [17,24,25]. The application of these methods has enabled a detailed understanding of how additives, molecular structures, and surfactants affect the stability and performance of the studied systems. The Langmuir technique offers a unique and powerful approach to investigating molecular interactions and the properties of lipids at phase boundaries, aspects that are often challenging to capture using conventional methods such as FTIR spectroscopy, gas chromatography, or rheological analyses. Unlike these traditional techniques, which primarily focus on analyzing chemical composition or bulk properties, Langmuir monolayer analysis enables an in-depth study of the dynamic and structural characteristics of lipid monolayers at the gas/liquid interface [26]. This includes detailed insights into the orientation, packing, and behavior of lipid molecules, which play a critical role in understanding the processes of oxidation and the long-term stability of lipids. The ability to directly observe these molecular interactions in real time offers a more nuanced understanding of lipid behavior, especially under different environmental conditions such as exposure to heat or oxygen. Integrating this method into the study of vegetable oils represents a significant advancement, extending analytical capabilities beyond traditional approaches that primarily focus on chemical and physicochemical changes in lipid components. By highlighting molecular dynamics at the air/liquid interface, Langmuir monolayers provide a more comprehensive framework for evaluating the stability and oxidative processes in vegetable oils, which is crucial for their optimization in various industrial applications.

In this study, the Langmuir monolayer method and oscillating barriers were applied to monitor oxidation changes of some vegetable oils during an accelerated storage test. By employing the Langmuir monolayer method, the research analyzes the molecular interactions, packing, and orientation of lipid molecules at the air/liquid interface at different stages of oil oxidation, providing insights into their mechanical and dynamic properties. This investigation aims to highlight the significance of these methods in food technology and their potential applications in various industrial processes, including the optimization of the quality and shelf life of vegetable oils. The findings of this research contribute to a better understanding of the impact of oxidation processes on oils’ stability, potentially influencing the development of new strategies for their storage and utilization in the food industry.

## 2. Results and Discussion

In this study, six cold-pressed oils were subjected to an accelerated storage test at 60 °C. This test allowed for the evaluation of the oils’ stability under thermal stress, which can lead to the degradation of unsaturated fatty acids and, consequently, a reduction in oil quality. The tested oils were milk thistle oil (MTSO), evening primrose seed oil (EPSO), flaxseed oil (FSO), camelina sativa seed oil (CSSO), black cumin seed oil (BCSO), and pumpkin seed oil (PSO). To investigate the thermodynamic behavior of both fresh oils and those subjected to thermal treatment, they were compressed at the air/water interface, compression isotherms were recorded, and the modulus of surface viscoelasticity was calculated.

### 2.1. The Fatty Acid Profile

The fatty acid profile (FAME) of the studied oils, presented in Table 1, was analyzed using Ultra-Performance Liquid Chromatography (UPLC). The levels of major fatty acids, including saturated fats (SFA), monounsaturated fats (MUFA), and polyunsaturated fats (PUFA), were determined. EPSO showed a high PUFA content of 70.54%, primarily from linoleic acid (C18:2), along with MUFA from oleic acid (C18:1) at 17.32%, and an SFA content of 12.05%. A similar extremely high amount of C18:2 in EPSO was found in [27]. MTSO exhibited 62.09% PUFA, mainly due to linoleic acid (47.7%), and MUFA from oleic acid (25.87%), which is comparable with other research [28], and had an SFA content of 9.67%. FSO also demonstrated significant PUFA levels at 61.16%, mainly from α-linolenic acid (C18:3, n-3) (46.73%), and MUFA from oleic acid (20.73%), while SFA accounted for 17.87%. A slightly higher content of α-linolenic acid in FSO was found in [15], simultaneously with nearly equal contents of C18:0, C18:1, and C18:2. BCSO showed a sustainable composition, with oleic acid at 26.42% and an SFA content of 12.92%. PSO contained the highest amount of SFA at 21.18%, with a total PUFA content of 53.24%, mainly from linoleic acid (52.41%). These results agree with [15], who reported that oleic and linoleic acids are the primary fatty acids comprising BCSO and PSO. CSSO was characterized by 59.45% PUFA, primarily α-linolenic acid (52.70%), and 20.52% MUFA, mainly oleic acid, with an SFA content of 12.31%, consistent with values reported in previous studies [29]. The analyzed oils exhibited a diverse range of FAME, with a high PUFA content, particularly noted in EPSO, MTSO, and FSO, primarily due to linoleic acid and α-linolenic acid. In contrast, PSO displayed relatively comparable proportions of MUFA and SFA, simultaneously containing the highest amount of SFA among all the oils.

### 2.2. Specific Extinction Coefficients

Oil oxidation is a complex process which involves autoxidation and photo-oxidation. The rate of oxidation depends on a variety of factors, including the chemical composition of oil, temperature, or exposure to light. Also, the presence of oxygen accelerates the oxidation process in oils [30,31]. Antioxidants present inherently in vegetable oils, such as tocopherols and phenolic compounds, are able to reduce the speed of oil oxidation at room temperature. The same rules apply to synthetic antioxidants, such as BHA and propyl gallate, added to oils to improve their stability. However, the effectiveness of antioxidants varies with the composition of fat and decreases at higher temperatures [30,32]. The main changes in stored oil occur due to the presence of a double carbon bond in the fatty acid, whose sensitivity to oxidation increases exponentially as a function of the number of double bonds per fatty acid molecule. Therefore, PUFAs are prone to oxidation much more than MUFAs or SFAs [33].

The oxidative stability of the tested cold-pressed oils was evaluated by monitoring the *K*_232_ and *K*_268_ extinction coefficients, which reflect the formation of primary and secondary oxidation products, respectively. Higher values of *K*_232_ and *K*_268_ indicate a more advanced oxidation stage. The *K*_232_ and *K*_268_ values for the tested cold-pressed oils are summarized in Table 2. These values were determined using Equation (1), based on absorbance measurements at wavelengths of 232 nm and 268 nm. Over the 21-day storage period, each oil type displayed distinct patterns in these values, highlighting variations in oxidative stability and susceptibility to degradation.

The extinction coefficient *K*_232_, indicating the presence of conjugated dienes and primary oxidation products, noticeably increased in all analyzed oils during the storage period. In the case of MTSO, a steady rise in *K*_232_ values was observed, from 6.02 to 35.21 (an increase of 5.9 times), after 21 days of heating, suggesting the high susceptibility of this oil to the early stages of oxidation. EPSO exhibited a larger increase in the *K*_232_ value, rising from 7.60 on day 0 to 109.70 (an increase of 14.4 times) on day 18. This pattern indicates rapid primary oxidation. *K*_232_ values for FSO increased with a similar growth rate, from 1.80 on day 0 to 26.70 on day 18. However, after 21 days, an increase in the *K*_232_ value of 32.2 times was noted, suggesting that FSO undergoes rapid primary oxidation, likely due to its specific fatty acid composition. CSSO showed a moderate increase, with *K*_232_ values rising from 1.60 on day 0 to 22.70 on day 21 (an increase of 14.2 times), indicating a relatively stable level of susceptibility to oxidation. PSO and BCSO exhibited the smallest increases in *K*_232_ values, from 7.50 to 16.00 (an increase of 2.2 times) and 5.60 to 8.00 (an increase of 1.4 times), respectively, indicating higher resistance to primary oxidation, likely due to the presence of stabilizing compounds or a more resilient FAME.

The values of the *K*_268_ parameter, which is associated with conjugated trienes and secondary oxidation products, at the first stage of thermal treatment slightly increased for most oils. A significant increase in this parameter occurred in the middle stage of the test, reflecting the more efficient transition from primary to secondary oxidation. The *K*_268_ value for MTSO increased from 0.31 on day 0 to 3.71 (an increase of 12.0 times) on day 21, confirming the progressive accumulation of secondary oxidation products, consistent with observed increases in *K*_232_. EPSO exhibited the highest *K*_268_ values among the tested oils, rising from 0.07 to 10.46 (an increase of 149.4 times) on day 18. This trend indicates rapid accumulation of secondary oxidation products, which reflects the lower oxidative stability of EPSO compared to other oils. A slightly lower growth rate of *K*_268_ was observed for CSSO, from 0.03 to 2.69 (an increase of 89.7 times). The *K*_268_ value for FSO increased moderately, from 0.14 to 2.65 (an increase of 18.9 times), reflecting moderate formation of secondary oxidation products and suggesting balanced oxidative stability. For PSO and BCSO, only a slight variation in *K*_268_ values was observed, highlighting their oxidative resistance and suggesting that these oils are better suited for long-term storage without significant degradation.

EPSO demonstrated greater susceptibility to both primary and secondary oxidation, as evidenced by the rapid increases in *K*_232_ and *K*_268_ values associated with the highest PUFA content. On the contrary, PSO and BCSO, containing the lowest PUFA levels, exhibited greater resistance to oxidative degradation, maintaining stable *K*_232_ and *K*_268_ values throughout the storage period. While FSO, MTSO, and CSSO contained comparable PUFA amounts, they differed in oxidation rates. FSO contained almost 47% of C18:3, n-3, and exhibited the fastest oxidation rate among them. Although MTSO and CSSO also contain higher levels of linoleic acid and α-linolenic acid, they are at the same time, as indicated by other studies, rich in natural antioxidants such as tocopherols (especially α-tocopherol in the case of MTSO) [28], which are capable of capturing peroxyl radicals [34]. Among all the tested oils, BCSO, especially rich in phenolic compounds [15,35], proved to be the most stable during storage. The high oxidative stability of BCSO, compared to other cold-pressed oils, has also been reported by other studies [15,36]. The obtained results indicate that the growth rate of *K*_232_ and *K*_268_ values mostly reflected the susceptibility of the studied oils to oxidation related to the content of PUFAs. Similarly, Abdulkarim et al. [14] found that the content of conjugated dienes was significantly influenced by the fatty acid composition of the oil, with levels of dienes and trienes rising as the proportion of unsaturated fatty acids increased.

### 2.3. Characteristics of Langmuir Monolayers of Fresh Oils

#### 2.3.1. Surface Pressure–Mean Molecular Area Isotherms

**Figure 1:**
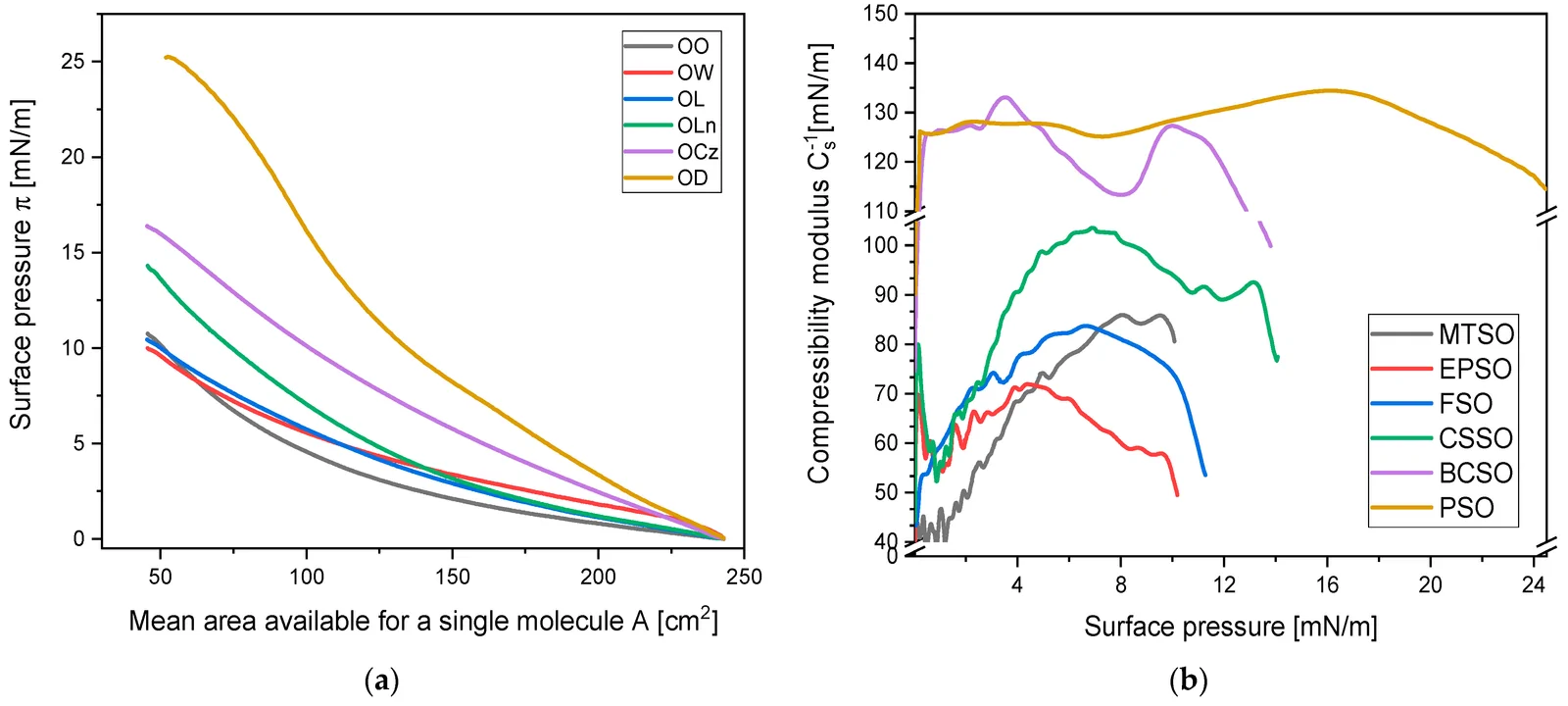
Compression π-A isotherms of Langmuir monolayers (**a**) and compression modulus–surface pressure dependences (**b**) for the tested oils.

During compression of the monolayer depending of molecules interactions it can occur in different thermodynamic phases named as: liquid-expanded (LE), liquid (L), liquid-condensed (LC) and the solid (S) state [37]. Surface pressure isotherms (π), as a function of the mean molecular area per oil molecule (A), were recorded during compression to provide detailed data on how fatty acid content affects the thermodynamic state and phase transitions of the formed oil monolayers (Figure 1a). To enable quantitative analysis and visualization of molecular interactions, the characteristic parameters of π-A isotherms, such as the extrapolated average surface area available to the molecule (A_EXP_), the average surface area available to the molecule at the collapse point (A_C_), and the surface pressure at the collapse point (π_c_), were determined and are presented in Table 3. A_EXT_ was determined by extrapolating the tangent to the first linear slope of the isotherm to π = 0.

The recorded oil compression isotherms significantly varied between samples. The studied oils were rich in oleic acid, linoleic acid, and α-linolenic acid (Table 1). Previous research has shown that the surface properties of plant oils are closely linked to their molecular composition, especially the content of individual fatty acids [19]. The isotherms recorded for MTSO, EPSO, and FSO, which contain higher levels of PUFAs, were less steep compared to the other tested oils. Additionally, it was observed that the isotherms display variable slopes as the mean molecular area decreases, in comparison to the initial stages of compression. This behavior is reflected in the changes in A_EXT_. That behavior corresponds to the phase transition of the monolayer to the LE phase. Depending on the tested compound, A_EXP_ occurred at different molecular area values. The highest A_EXT_ values were observed for PSO (174.7 cm^2^) and BCSO (182.4 cm^2^).

Additionally, it was noted that after the phase transition from the LE to LC phase, the isotherms for PSO, BCSO, and CSSO in the higher-surface-pressure region shifted towards smaller average molecular areas, while those for MTSO, EPSO, and FSO exhibited the opposite tendency and shifted towards larger areas. The phase transition of monolayers from the LE to LC phase is represented on the π-A isotherm by a plateau, whose value refers to the stability of the monolayer. The highest π_c_ value was achieved by PSO (25.3 mN/m), indicating that it formed the most stable layer among all the tested oils. Simultaneously, PSO contained the lowest proportion of unsaturated fatty acids. Differences in observed π_c_ values may result from the number of double bonds in the hydrocarbon chain. An increase in the unsaturation of aliphatic chains leads to reduced stability of the formed layers, which was observed in the studied oils. The orientation of oil molecules, which affected the layer behavior, largely depends on oil composition and the molecular structure of individual components. Previous studies have shown that the character of the recorded π-A isotherm strongly depends on the dominant acids in the studied mixtures [19]. Additionally, it was noted that an increase in the length of the apolar chain for molecules with the same number of double bonds affected the increase in the surface area per molecule, as previously observed in studies on fatty acids. The effect of the double bond directly influences the positioning of molecules at the water/air interface [23,24,38,39,40].

#### 2.3.2. The Compression Modulus and Surface Viscoelasticity Modulus

**Figure 2:**
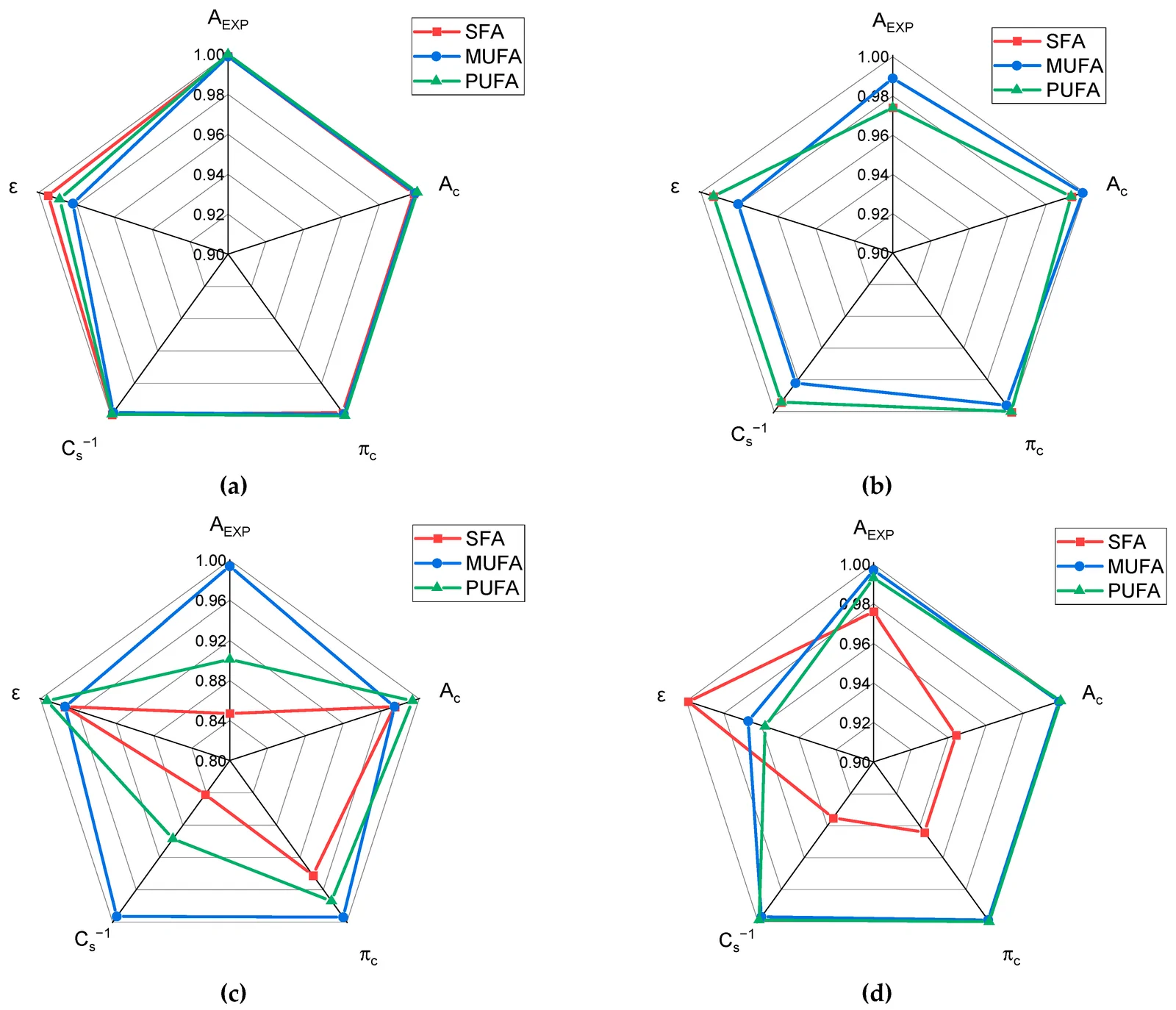

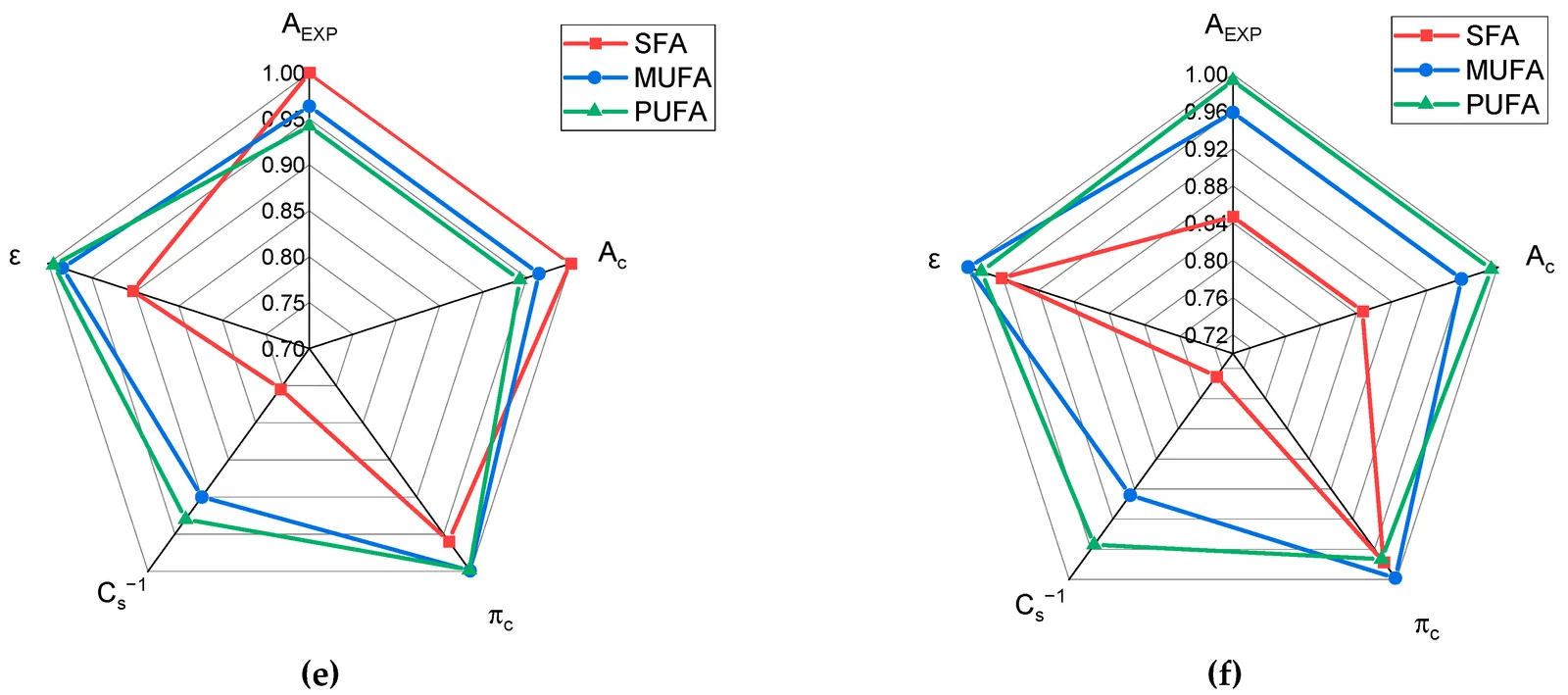
Correlation of fatty acid composition (FAME) with π-A isotherm parameters for different vegetable oils: (**a**) MTSO, (**b**) EPSO, (**c**) FSO, (**d**) CSSO, (**e**) BCSO, (**f**) PSO.

Based on the recorded π-A isotherms and the measurements of monolayer behavior under dynamic conditions, the compressibility modulus (C_S_^−1^) and surface viscoelasticity (ε) were determined using Equations (2) and (5), respectively. The analysis of these parameters provided additional information for characterizing the mechanisms of stability in lipid monolayers and their physicochemical properties. The relationships between the compression modulus C_S_^−1^ and surface pressure π (C_S_^−1^-π) presented in Figure 2b allowed for a detailed analysis of the phase state of compressed Langmuir monolayers. The determined values of C_S_^−1^ and ε are presented in Table 4.

The C_S_^−1^ modulus reflects how the monolayer responds to compression, indicating the elasticity and packing density of oil molecules at the air/water interface. Higher values of this modulus suggest a stiffer and more stable monolayer, indicative of a well-packed and condensed oil structure. In contrast, the ε modulus represents the viscoelastic properties of the monolayer, combining both elasticity (storage component) and viscosity (loss component), offering insight into the oil’s ability to withstand mechanical deformations. Higher ε values indicate stronger intermolecular interactions, suggesting that the oil monolayer is more resilient and can maintain its structural integrity under stress, which is often correlated with oils rich in saturated or monounsaturated fatty acids. The classification of the phase states of the Langmuir films was based on the criterion by Davies and Rideal [41]. According to this criterion, C_S_^−1^ values range from 12.5 to 50 mN/m for the LE phase, from 50 to 100 mN/m for the L phase, from 100 to 250 mN/m for the LC phase, and above 250 mN/m for the S state. The minima in the C_S_^−1^ versus π plots indicate the surface pressures at which phase transitions or significant molecular reorganization occurs. On the curves showing the dependence of C_S_^−1^ on π for the oil monolayers, distinct maxima corresponding to the LE and LC phases can be observed (Figure 1b). According to the adopted criterion, the maximum value of C_S_^−1^ indicates the state in which the layer was most compressed. The highest C_S_^−1^ values, exceeding 100 mN/m, were recorded for the PSO (138.5 mN/m), BCSO (136.4 mN/m), and CSSO (104.7 mN/m), indicating that they formed a layer in the LC phase. In contrast, for the remaining oils, the C_S_^−1^ value was within the range of 50–100 mN/m, which is characteristic of the L phase of the monolayer. Based on the recorded data, it is evident that PSO formed the most elastic monolayer during compression on the subphase surface. The observed decrease in C_S_^−1^ was due to an increase in the unsaturation of the hydrocarbon chain, which has the greatest impact on this parameter for the EPSO with the highest PUFA content (Table 1).

In the case of surface elasticity, the recorded ε values can be divided into three zones. In the first zone, when dynamic elasticity does not exceed 50 mN/m, long-range forces lead to the formation of a regular structure, with distances between particles larger than their diameter. Further oscillatory movement of the barriers causes these forces to be exceeded, and the particles become more closely packed. In this zone, the ε value can range from 50 to 250 mN/m. ε values within this range were observed for all tested oils. The third zone, corresponding to ε values above 250 mN/m, is characterized by high compression and a densely packed monolayer [42]. The ε values for all tested oils ranged from 68.9 to 122.3 mN/m, confirming that their viscoelastic properties fall within the second zone of surface elasticity. PSO and BCSO exhibited the highest surface viscoelasticity values (122.3 mN/m and 112.6 mN/m, respectively), suggesting that they formed the most elastic and closely packed monolayers under dynamic conditions. CSSO, with ε equal to 94.8 mN/m, formed a stable and elastic monolayer, although slightly less elastic compared to PSO and BCSO. MTSO and FSO showed ε values of approximately 74.2 mN/m and 70.3 mN/m, respectively, indicating that they formed less elastic monolayers while remaining within the characteristic range of the second zone. Finally, EPSO exhibited the lowest ε value (68.9 mN/m), indicating lower rigidity than the other oils but still sufficient elasticity to form a stable monolayer. For all tested oils, the ε values were similar to the C_S_^−1^, suggesting that the adsorbed molecules formed an elastic monolayer at the interface, which is crucial for their stability.

#### 2.3.3. Correlation Between π-A Isotherm Parameters and Fatty Acid Composition

In the analysis of correlations between FAME and parameters derived from oils’ Langmuir isotherms, significant variations in results were observed. These correlations suggest that fatty acid saturation markedly influences intermolecular interactions within monomolecular layers as well as the surface behavior of these fatty acids. Figure 2 presents a radar plot showing the correlation of SFAs, MUFAs, and PUFAs with the determined π-A isotherm parameters in the analyzed oils. 

Oils with higher SFA saturation exhibited strong correlations between fatty acid content and Langmuir parameters such as A_EXP_, A_C_, and π_c_, often approaching a value of 1. For example, in BCSO, SFA content correlations with A_EXP_ and A_C_ parameters reached a value of 1, which may indicate the high stability of these fatty acids within the monomolecular layer and their capacity to form well-organized structures. Similarly, for CSSO, correlations for A_C_ and π_c_ were 0.944, suggesting a strong link between fatty acid structure and surface interactions. In contrast, oils with higher levels of MUFAs and PUFAs, such as FSO and CSSO, demonstrated diverse correlations between fatty acid content and Langmuir parameters. For instance, for FSO, characterized by a low SFA and higher MUFA and PUFA content, strong correlations between them and A_EXP_ (equal to 0.847, 0.994, and 0.901, respectively) and π_c_ (equal to 0.942, 0.994, and 0.974, respectively) were found. Similarly, CSSO showed high correlations between PUFA content and A_EXP_ and ε, suggesting that higher fatty acid unsaturation may promote the formation of more dynamic surface structures. The ε parameter exhibited a lower correlation with FAME, especially for oils with higher PUFA contents, potentially indicating increased surface structure variability. For PSO and FSO, which contained higher levels of SFA, the ε correlations were stronger than those observed for BCSO and CSSO.

### 2.4. Analysis of Oxidized Oil Monolayers

#### 2.4.1. π-A Isotherms of Oils During Storage Test

**Figure 3:**
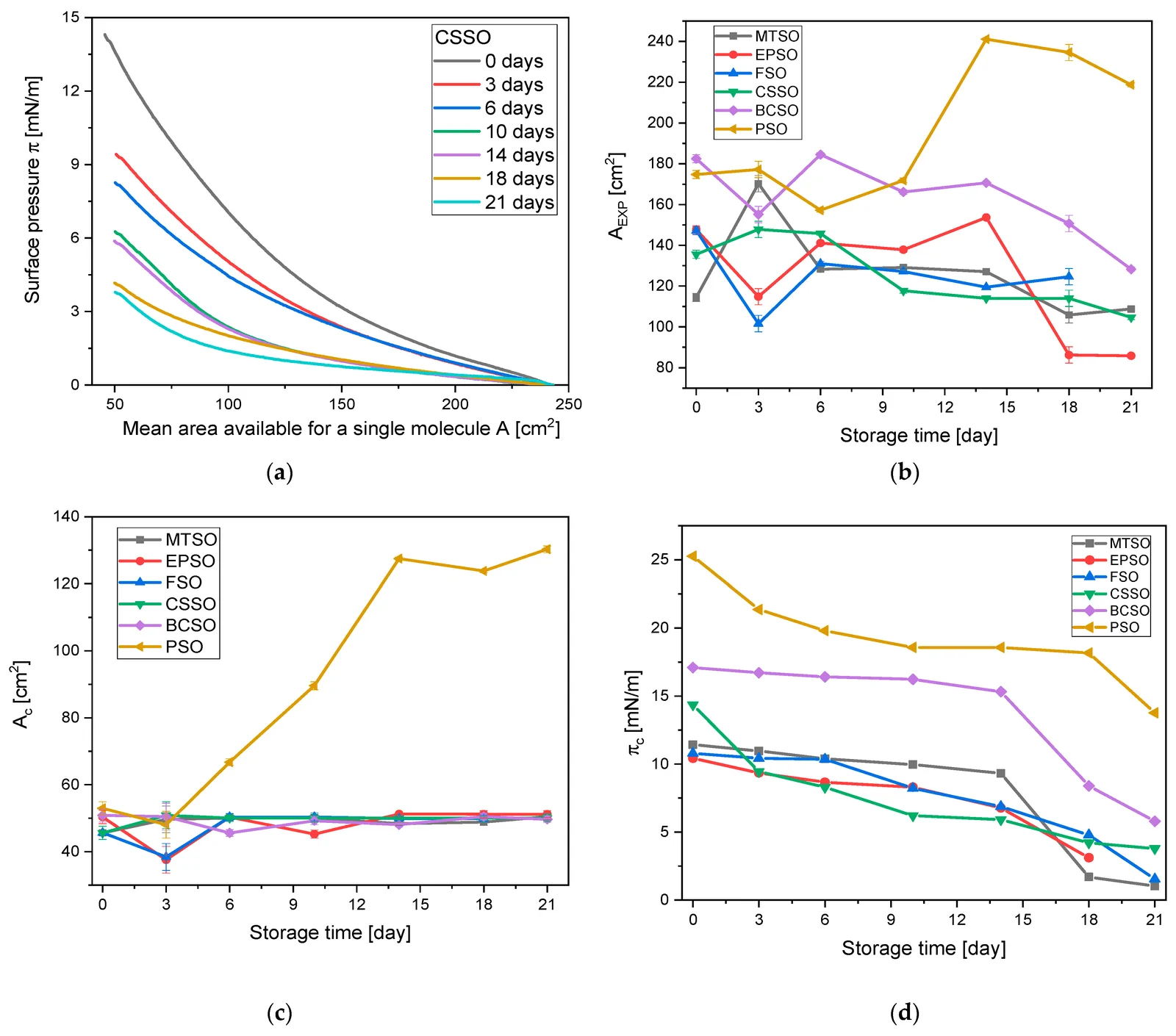
Compression isotherms of Langmuir monolayers for CSSO on different days of storage (**a**), dependences of the extrapolated mean molecular area (A_EXT_), (**b**) and the mean molecular area at the collapse point (A_C_) on the tested oil (**c**), as well as the dependence of the surface pressure at the collapse point (π_c_) on the tested oil (**d**).

The tested oils were stored at 60 °C for three weeks. Samples were taken for analysis on the 3rd, 6th, 10th, 14th, 18th, and 21st days of heating. An example of the recorded π-A isotherms for the oxidized CSSO during compression is presented in Figure 3a. The characteristic parameters determined from the π-A isotherms are shown in Figure 3b–d. During the conducted measurements of Langmuir monolayers of oxidized oils, changes in the shape of the π-A isotherm and variations in the values of A_EXP_, A_c_, and π_c_ were observed with storage time. The oxidation rate of all tested oils increased significantly after the 10th day (Table 2), which may indicate a critical threshold above which oxidative degradation accelerates. It was noted that oils with a higher content of PUFA exhibited a faster oxidation rate compared to others. This was evidenced by a significant decrease in the determined parameters, indicating a substantial loss of oil quality and susceptibility to oxidative damage. Although MTSO demonstrated higher oxidative stability compared to CSSO, FSO, and EPSO (see Section 2.2), the monolayers observed for CSSO exhibited a gradual shift in π_c_, indicating that despite the ongoing oxidation process, the oil preserved its structural integrity. These results, obtained for CSSO, which contains more SFA and fewer unsaturated fatty acids compared to MTSO, demonstrate that the fatty acid composition is the dominant factor in the oil monolayer behavior, whereas PSO exhibited a more stable monolayer and fewer changes in surface properties compared to the aforementioned oils. PSO is characterized by a lower content of PUFAs, which contributed to the observed improved oxidative stability. As we can see from Table 2, BCSO demonstrated the highest resistance to oxidation, which is reflected in the stability of the Langmuir monolayer properties. Despite the observed decrease in key parameters, the rate of its decrease was significantly slower compared to other oils (Figure 3).

Oxidation of oils induces a series of chemical transformations in the fatty acid molecules, leading to the formation of new functional groups, such as carbonyl, hydroxyl, and peroxide groups. These newly introduced groups significantly alter the polarity of the molecules. As the oxidation process progresses, the increased polarity of the fatty acid molecules influences their interactions within the Langmuir monolayer, affecting the packing and stability of the monolayer. In the initial stages of oxidation, the incorporation of polar groups reduces the intermolecular interactions, which manifests as changes in the parameters derived from the π-A isotherms, such as a decrease in surface pressure (π). Over time, as oxidation advances, the hydrocarbon chains begin to fragment, and oxidation products, such as aldehydes and ketones, are formed. These smaller, more polar oxidation products further weaken the molecular interactions within the monolayer. As a result, the surface pressure continues to decrease, and the monolayer structure becomes increasingly destabilized. The oil molecules become more susceptible to separation, leading to a less organized and more fluid monolayer with decreased stability. This process ultimately results in a significant alteration of the monolayer’s structural integrity, reflecting the degradation of the oil’s quality due to the oxidative process.

#### 2.4.2. Modulus of Compressibility and Surface Viscoelasticity

**Figure 4:**
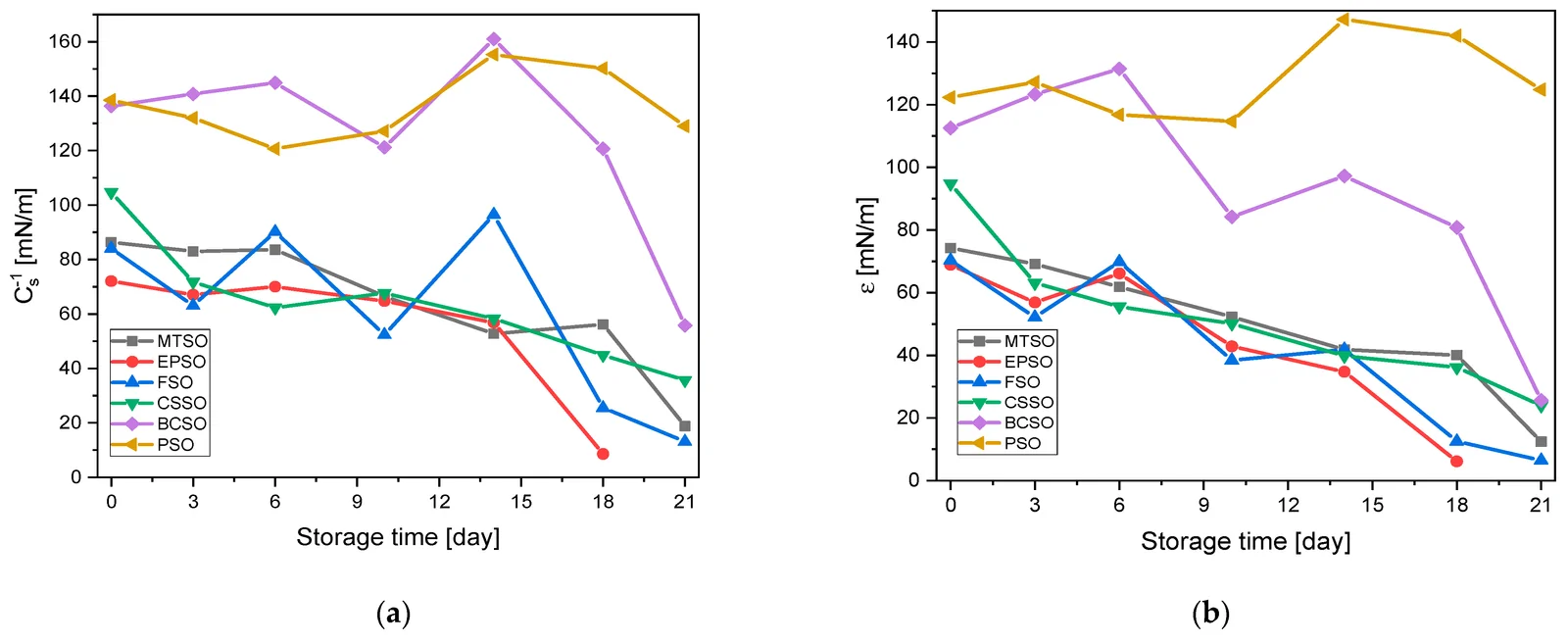
Dependence of the compressibility modulus C_S_^−1^ (**a**) and the viscoelastic modulus ε (**b**) on time for the tested oil.

The C_S_^−1^ and ε modulus of lipid monolayers were studied under dynamic conditions during a 21-day storage period (Figure 4). For unheated oils, the highest values of C_S_^−1^ were observed for PSO, BCSO, and CSSO (Table 4), which suggests that these oils formed well-packed and stable monolayers. High values of C_S_^−1^ indicate the rigidity and resistance of these monolayers to compression.

Over time, the C_S_^−1^ values, similarly to the π_c_ parameter, systematically decreased for all oils, reflecting the deterioration of monolayer stability (Figure 4a). The most pronounced decrease was observed for FSO and EPSO. After 21 days, the C_S_^−1^ values decreased to 13.1 mN/m and 8.6 mN/m, respectively, corresponding to the LE and L phases. The reduction in C_S_^−1^ values suggests a loosening of the monolayer structure, likely due to oxidative processes, particularly affecting oils rich in PUFAs. In contrast, PSO and BCSO, despite a noticeable reduction in C_S_^−1^, maintained relatively high values even after 21 days of thermal storage (128.9 mN/m and 155.3 mN/m, respectively). This confirmed that these oils’ monolayers exhibit greater structural stability and resistance to oxidation.

At the start of the storage test, the highest ε values were recorded for PSO, BCSO, and CSSO (Table 4). The elevated ε values indicate robust intermolecular interactions and significant elasticity of the monolayers, enabling them to preserve their structural integrity even under mechanical stress. Similarly to C_S_^−1^, the ε values gradually declined over the course of storage, pointing to the degradation of the lipid monolayer structure. The lowest ε values were observed for FSO and EPSO. After 21 days, they reached 6.5 mN/m and 6.2 mN/m, respectively, indicating a substantial loss of elasticity and monolayer stability. This aligns with the C_S_^−1^ results, suggesting that these oils undergo rapid oxidation, weakening their mechanical properties. In contrast, PSO and BCSO retained high monolayer elasticity even after 21 days (124.8 mN/m and 25.6 mN/m, respectively). The high ε values for these oils indicate that they are more resistant to aging and oxidation.

During the accelerated storage test, oxidative degradation of oils occurred, and simultaneously, a decrease in both parameters’ values was observed. The values of Langmuir monolayer parameters for some oils, such as PSO, initially increased in the early oxidation stages, leading to the formation of products with stronger intermolecular interactions and molecular rearrangement. Such degradation leads to the breakdown of fatty acids, resulting in less stable and more disorganized monolayers. Oils rich in PUFAs, such as FSO and EPSO, tend to show a significant reduction in both parameters over time, indicating their greater susceptibility to oxidation. In contrast, oils exhibiting greater oxidative stability, with a higher content of SFAs (e.g., PSO or BCSO), maintained higher values of both parameters.

As was seen from our results, analyzing the C_S_^−1^ and ε modulus could be used for predicting the durability and oxidative stability of vegetable oils. Oils exhibiting higher values of both parameters are more likely to retain their quality over a longer period, while those with lower values are more prone to rancidity. C_S_^−1^ and ε provide essential insights into the structural and oxidative stability of vegetable oils at the molecular level.

#### 2.4.3. Correlation Between Isotherm π-A Parameters of Oxidized Oils and *K*_232_ and *K*_268_

**Figure 5:**
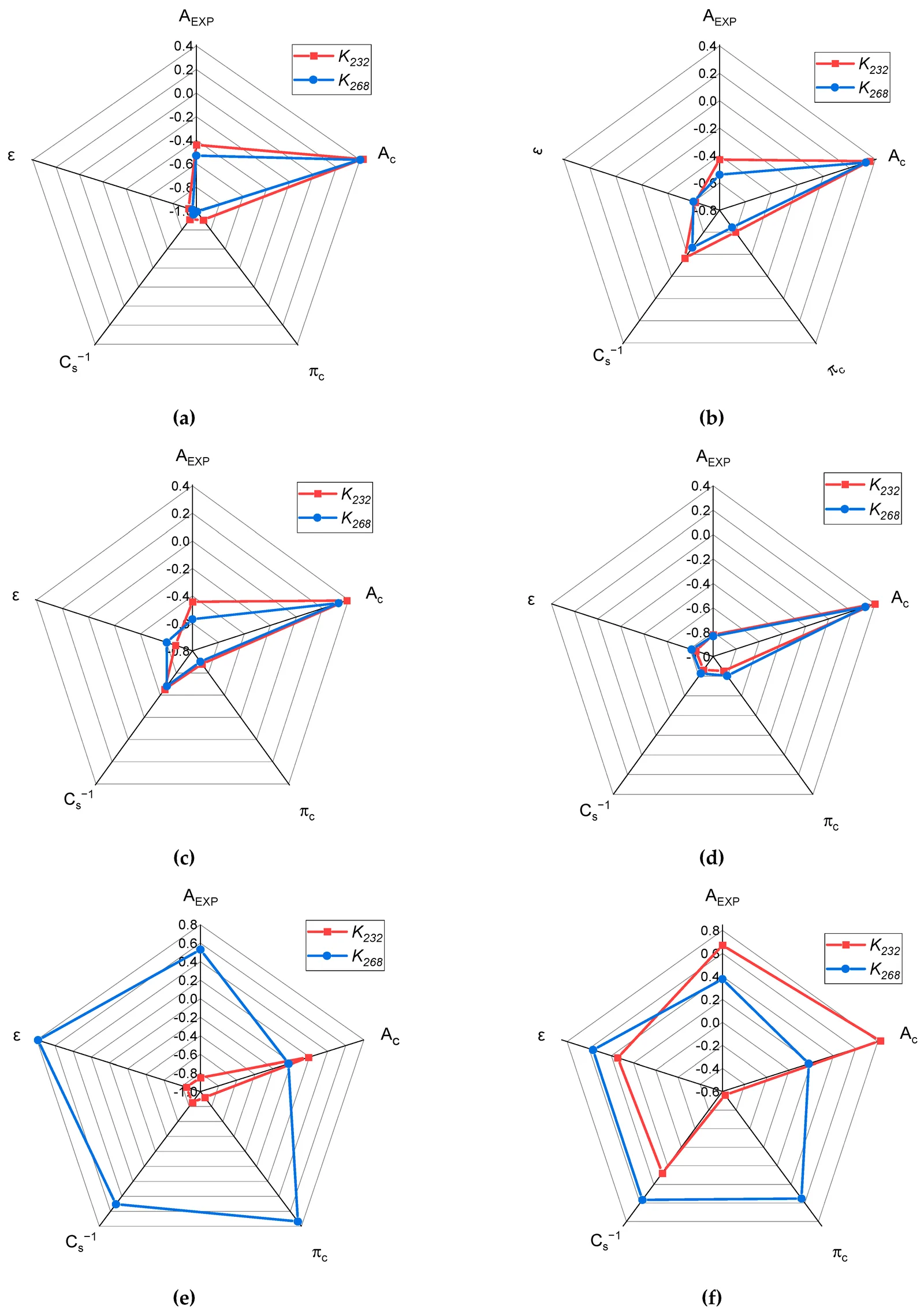
Correlation of *K*_232_ and *K*_268_ extinction coefficients with π-A isotherm parameters for different vegetable oils: (**a**) MTSO, (**b**) EPSO, (**c**) FSO, (**d**) CSSO, (**e**) BCSO, (**f**) PSO.

In analyzing the correlations between the oxidation indices *K*_232_ and *K*_268_ and the parameters derived from π-A isotherms for various vegetable oils, specific relationships were observed that indicate how oxidation processes impact intermolecular interactions and the surface stability of monomolecular films (Figure 5). These indices facilitated the assessment of oxidative stability in the studied oils during a 21-day accelerated storage test. The study of correlations between *K*_232_/*K*_268_ and Langmuir isotherm parameters (A_EXP_, A_C_, π_c_, C_S_^−1^, and ε) enabled an in-depth analysis of how the physicochemical properties of monolayer films influence each oil’s susceptibility to oxidation processes.

The most pronounced relationship was observed between the oxidation indices and π_c_. Negative correlations between the *K*_232_ index and π_c_ are particularly strong for MTSO (−0.904), CSSO (−0.852), and BCSO (−0.922), suggesting that increased susceptibility of an oil to oxidation is associated with a reduction in monolayer pressure. The reduced π_c_ may result from a decline in intermolecular interactions and weakened monolayer cohesion due to oxidation. The *K*_268_ index, reflecting a more advanced stage of oxidation, also showed strong negative correlations with π_c_, especially for MTSO (−0.991) and CSSO (−0.803). This observation suggests that as oxidation deepened, the monolayer pressure decreased, which may be related to a higher content of oxidized unsaturated fatty acids that less effectively supported the film structure.

Another important correlation was the negative relationship between the oxidation indices and the C_S_^−1^ parameter, which reflects the rigidity of the film. For oils with higher susceptibility to oxidation, such as MTSO (−0.909) and CSSO (−0.864), higher *K*_232_ values correlated with a reduction in monolayer elasticity. This result suggests that oxidation of the oil caused destabilization of the layer, making it less rigid.

For the *K*_268_ index, the correlation with C_S_^−1^ was also strong and negative, indicating that the oxidation process further weakens the monolayer structure. Similarly, negative correlations were observed for the *K*_232_ and *K*_268_ indices and the ε parameter. The results indicated a reduced ability of the monolayer to expand under oxidation, which may be indicative of weakened film flexibility. The correlations for ε with *K*_232_ were high, especially for MTSO (−0.934), while for the *K*_268_ index, the correlations were also significant, suggesting a further restriction in flexibility during advanced oxidation.

For the A_EXP_ parameter, the *K*_232_ and *K*_268_ indices showed negative correlations, indicating that oxidation leads to a decrease in the effective monolayer surface area, which could result from the aggregation of oxidation products on the surface. This correlation was particularly strong for CSSO (−0.821 and −0.832 for *K*_232_ and *K*_268_, respectively), which may be due to the higher content of PUFAs. Among the oils studied, PSO and BCSO showed higher A_C_ values and lower correlations with the *K*_232_ and *K*_268_ parameters, indicating their greater oxidative stability.

## 3. Materials and Methods

### 3.1. Materials

The studied material consisted of six cold-pressed vegetable oils derived from evening primrose seeds (*Oenothera paradoxa*)—EPSO; milk thistle seeds (*Silybum marianum*)—MTSO; pumpkin seeds (*Cucurbita oleo*)—PSO; flax seeds (*Linum usitatissimum* L.)—FSO; camelina sativa seeds (*Camelina silvestris*)—CSSO; and black cumin seeds (*Nigella sativa*)—BCSO. These oils were obtained in collaboration with SemCo (SemCo Sp. z o.o. Sp.k., Szamotuły, Poland). To assess the oxidative stability of the selected oils, an accelerated storage test under elevated temperature was conducted (Schaal oven test). The oils were placed in an oven heated to 60 °C to simulate oxidative aging processes and accelerate oxidation reactions. In this condition, one day at 60 °C is equivalent to one month at room temperature [43]. Samples were taken for analysis at the following time intervals: after 3, 6, 10, 14, 18, 21, and 24 days of storage.

### 3.2. Solvents

Methanol (CH_3_OH), used for fatty acid extraction and methylation in the FAME analysis, was obtained from POCH, Lublin, Poland. Chloroform (CHCl_3_), applied in the Langmuir monolayer experiments and for cleaning the Langmuir trough, was also purchased from POCH, Lublin, Poland (purity 99.8%). Pentane (C_5_H_12_), used for lipid extraction after neutralization, was supplied by Sigma-Aldrich, Saint Louis, USA. n-Hexane (C_6_H_14_), used in the methylation process and lipid extraction, was also sourced from Sigma-Aldrich, Saint Louis, USA. Sulfuric acid (H_2_SO_4_), used in the methanol/sulfuric acid mixture for methylation, was provided by Chempur, Piekary Śląskie, Poland. Isooctane (2,2,4-trimethylpentane) was obtained from POCH, Lublin, Poland. These high-purity solvents were essential for the accurate extraction, preparation, and analysis of the oils.

### 3.3. Methods

#### 3.3.1. Fatty Acid Profile (FAME) Analysis

The fatty acid profile (FAME) was determined in the analyzed oils by the Ultra-Performance Liquid Chromatography (UPLC) technique. Fatty acids were extracted by a method described by Stuper-Szablewska et al. [44]. Samples containing 100 mg of oil were placed into 17 mL culture tubes, suspended in 2 mL of methanol, treated with 0.5 mL of 2M aqueous sodium hydroxide, and tightly sealed. The culture tubes were then placed within 250 mL plastic bottles, tightly sealed, and placed inside a microwave oven (Model AVM 401/1 WH; Whirlpool, Bromma, Sweden) operating at 2450 MHz and 900 W maximum output. Samples were irradiated (370 W) for 20 s and, after approx. 5 min, for an additional 20 s. After 15 min, the contents of the culture tubes were neutralized with 1M aqueous hydrochloric acid; 2 mL MeOH was added and extraction with pentane (3–4 mL) was carried out within the culture tubes. The combined pentane extracts were evaporated to dryness in a nitrogen stream. In the next step, extracts were methylated using a mixture of anhydrous methanol and sulfuric acid (1:5, *v*/*v*). The extract containing lipids was added with 0.5 mL of methanol followed by an addition of a 0.15 mL methanol/sulfuric acid mixture (1:5, *v*/*v*). The samples were heated at 70 °C for 15 min. After the solution had been cooled, 0.5 mL of n-hexane was added, followed by the addition of sufficient water to form two layers. The upper hexane layer was removed and analyzed on an Aquity H class UPLC system equipped with a Waters Acquity PDA detector (Waters, Miford, CT, USA). Chromatographic separation was performed on an Acquity UPLC^®^ BEH C18 column (150 mm × 2.1 mm, particle size 1.7 μm) (Waters, Wexford, Ireland). The elution was carried out on a gradient using the following mobile phase composition: A: acetonitrile; B: 2-propanol; flow 0.17 mL/min. Compounds were identified based on a comparison of retention times of the examined peak with that of the standard and by adding a specific amount of the standard to the tested sample and repeated analyses.

#### 3.3.2. UV–Vis

The oxidative stability of six oils was evaluated by calculating the specific absorbance coefficients (*K*_232_, *K*_268_) of the oils, following the method described by AOCS [45]. The samples were diluted with isooctane, and the absorbance at wavelengths of 232 nm and 268 nm was measured in a 1 cm path-length cell using a Shimadzu UV-1201 spectrophotometer (Kyoto, Japan). The final values were calculated using Equation (1):

(1) $$K_{\lambda }=\frac{A_{\lambda }}{\left(c\cdot s\right)}$$

where *K_λ_*—the specific absorbance coefficient at wavelengths of 232 or 268 nm; *A_λ_*—the absorbance; *c*—the concentration of oil in the solvent (g/100 mL); and *s*—the path length (cm). Each sample was analyzed in triplicate, and the final result was the average of the three measurements for each sample.

#### 3.3.3. Langmuir Monolayer Method

Langmuir monolayers made from selected plant oils were compressed using two symmetrically driven hydrophilic barriers in a Langmuir trough (KN 2002, KSV NIMA, Helsinki, Finland) made of Teflon. The trough had a surface area of 273 cm^2^ and a subphase volume of 176 mL. Before the experiments, the trough was thoroughly cleaned with methanol and chloroform (a purity of 99.8%, POCH, Lublin, Poland). Ultrapure deionized water with a resistivity of 18.2 MΩ∙cm from the Milli-Q system was used as the subphase. The water surface filling the Langmuir trough was cleaned using a standalone aspirator pump until the change in surface pressure after maximal barrier displacement was below 0.1 mN/m. A precise amount of the sample material was immediately applied dropwise onto the clean subphase using a high-precision microsyringe (Hamilton, Reno, NV, USA). The compression of the monolayers was carried out at a barrier movement speed of 5 mm/min, following a 10 min period for chloroform evaporation. During compression, the surface pressure, π, was measured using a porous platinum Wilhelmy plate suspended on an electronic balance with an accuracy of 0.01 mN/m, as a function of the mean molecular area per molecule, A. Each π-A isotherm was recorded at least three times to confirm its reproducibility. The measurements were reproducible within an error of ±0.02 cm^2^.

During measurements, a π-A isotherm was recorded, showing the changes in surface pressure (π) as a function of the area per single molecule (A) [19]. Surface pressure is defined as the difference between the surface tension of the carrier phase before adding the test substance (σ_0_) and the surface tension after the monolayer has formed (σ). Equation (2) describes the relationship for surface pressure (π) [46]:

(2) $$\pi =\sigma_{0}-\sigma ,$$

Changes in surface pressure are associated with changes in the area per individual molecule, which affects the fluidity of the monolayer and its elasticity. The compressibility modulus C_S_^−1^, which measures the layer’s elasticity, is defined by the following Equation (3) [41,46]:

(3) $$C_{s}^{-1}=-A{\left[\frac{d\pi }{dA}\right]}_{T},$$

where A—area per individual molecule; π—surface pressure.

#### 3.3.4. Oscillatory Dilational Rheology

The dilational viscoelasticity of Langmuir monolayers was studied using the oscillating barrier method. In this experiment, the surface pressure response to small-amplitude sinusoidal variations in surface area was measured. The monolayers were initially compressed to the surface at a pressure of π_c_ and left to equilibrate for 20 min. Then, the barriers began oscillating, inducing small (1%) changes in the area available for the Langmuir monolayers. The experiments were conducted over a frequency range from 0.1 to 1 mHz, with at least 10 oscillation cycles recorded for each frequency. A 60 s interval was maintained between successive oscillation cycles. In the oscillating barrier method, the barriers are set into oscillatory motion with a specific frequency and amplitude. The system’s response to stress is measured as a change in surface pressure, *σ* = π(t) − π_0_, and monitored over time. According to this method, a harmonic perturbation of the surface with a specific amplitude occurs on the compressed monolayer through the barriers at a controlled angular frequency [21,47]:

(4) $$A\left(t\right)=A_{0}\left(1+∆usin\left(\omega t+\varphi_{u}\right)\right),$$

where A_0_—average surface area available to a single molecule at the moment of surface pressure increase; Δu = ΔA/A_0_—amplitude of the relative surface deformation; u(t) = (A(t) − A_0_)/A_0_; n = 2πv is the angular frequency and φ_u_ takes into account the possible onset phase.

The surface viscoelastic modulus ε and the phase *φ* of the complex dilational viscoelastic modulus can be expressed as functions of the measured values Δσ, Δu, φ_u_ and φ_π_ as a function of different frequencies [48,49]:

(5) $$\varepsilon =\frac{∆\sigma }{∆u},$$

Dilational interfacial rheology can be studied through various types of deformations, such as transient or harmonic surface changes. The relationship for dilational viscoelasticity ε is given by the following equation [50,51]:

(6) $$\varepsilon =-A\left[\frac{d\pi }{dA}\right]$$

### 3.4. Statistical Analysis

The measurements were performed in triplicate (unless otherwise stated in the method description). The values of the analyzed variables are presented as mean ± standard deviation (SD). The obtained results were statistically processed using OriginPro Software for Windows, Version 2023b (OriginLab Corporation, Northampton, MA, USA). The significance of differences between groups was determined based on one-way analysis of variance (ANOVA) and Tukey’s post hoc test (*p* < 0.05). Differences between results for respective oils marked with the same letter in the same row are statistically insignificant (*p* < 0.05). Relationships between variables were analyzed using Pearson’s correlation coefficient (r) in OriginPro, with values of *p* < 0.05 considered statistically significant.

## 4. Conclusions

This study demonstrated that the oxidative stability and surface properties of cold-pressed vegetable oils were directly linked to their fatty acid profiles. The use of the Langmuir monolayer technique enabled a sophisticated analysis of the molecular behavior of oils at the air/water interface, allowing for detailed insights into how lipid structure impacts surface stability and susceptibility to oxidation under various conditions.

The correlation results confirmed that the Langmuir monolayer technique is an effective tool for assessing the oxidative stability and surface properties of vegetable oils. Parameters such as molecular area, surface pressure, and elasticity modulus can serve as indicators of oil quality and storage viability. Studies of monolayer phase transitions—from the liquid-expanded to the liquid-condensed phase—revealed a relationship between fatty acid structure and the monolayer’s ability to maintain stability. Oils rich in PUFAs tend to undergo phase transitions more readily, decreasing their stability, while oils with higher SFA content retain phase stability for longer.

In summary, this study provided valuable insights into the complex relationships between the lipid composition and oxidative stability of vegetable oils. Using the Langmuir monolayer method allowed for the precise monitoring of molecular structural changes in response to oxidation processes, which can serve as a strong foundation for developing new methods to improve the quality and extend the shelf life of vegetable oils.

## Figures and Tables

**Table 1:** The fatty acid profile (FAME) in investigated oils.

Fatty Acid [%]	MTSO	EPSO	FSO	CSSO	BCSO	PSO
C14:0	1.76 ± 0.13 ^a^	nd	nd	nd	0.16 ± 0.01 ^c^	0.28 ± 0.01 ^b^
C16:0	nd	8.74 ± 0.05 ^d^	11.59 ± 0.17 ^b^	8.46 ± 0.05 ^d^	11.20 ± 0.10 ^c^	12.60 ± 0.02 ^a^
C16:1	0.55 ± 0.06 ^c^	nd	nd	2.70 ± 0.10 ^a^	1.20 ± 0.15 ^b^	0.55 ± 0.01 ^c^
C18:0	4.43 ± 0.04 ^c^	3.31 ± 0.10 ^d^	5.73 ± 0.05 ^b^	0.36 ± 0.04 ^e^	0.33 ± 0.04 ^e^	8.30 ± 0.02 ^a^
C18:1	25.87 ± 0.45 ^a^	17.32 ± 0.25 ^d^	20.73 ± 0.21 ^c^	20.52 ± 0.30 ^c^	26.42 ± 0.87 ^a^	24.05 ± 0.31 ^b^
C18:2	47.70 ± 0.42 ^d^	70.30 ± 0.20 ^a^	14.28 ± 0.18 ^e^	6.75 ± 0.05 ^f^	55.5 ± 0.36 ^b^	52.41 ± 0.17 ^b,c^
C18:3, n-3	14.23 ± 0.13 ^c^	0.24 ± 0.04 ^f^	46.73 ± 0.18 ^b^	52.70 ± 0.20 ^a^	1.21 ± 0.10 ^d^	0.83 ± 0.02 ^e^
C18:3, n-6	0.16 ± 0.01 ^a^	nd	0.15 ± 0.01 ^a^	nd	nd	nd
C20:0	3.48 ± 0.04 ^a^	nd	0.55 ± 0.01 ^c^	3.49 ± 0.04 ^a^	1.23 ± 0.15 ^b^	nd
C20:1	0.18 ± 0.01 ^b^	nd	0.17 ± 0.01 ^b^	0.18 ± 0.01 ^b^	0.33 ± 0.05 ^a^	nd
SFA	9.67 ± 0.02 ^e^	12.05 ± 0.04 ^d^	17.87 ± 0.02 ^b^	12.31 ± 0.01 ^d^	12.92 ± 0.01 ^c^	21.18 ± 0.20 ^a^
MUFA	26.42 ± 0.32 ^a^	17.32 ± 0.23 ^d^	20.73 ± 0.05 ^c^	23.22 ± 0.31 ^b^	27.62 ± 0.42 ^a^	24.61 ± 0.21 ^b^
PUFA	62.09 ± 0.71 ^b^	70.54 ± 0.32 ^a^	61.16 ± 0.56 ^b^	59.45 ± 0.81 ^b,c^	56.71 ± 0.42 ^c,d^	53.24 ± 0.32 ^d^

Explanatory notes: The data in the table are presented for investigated oils: milk thistle oil (MTSO), evening primrose seed oil (EPSO), flaxseed oil (FSO), camelina sativa seed oil (CSSO), black cumin seed oil (BCSO), and pumpkin seed oil (PSO). The data in the table are presented as the mean ± standard deviation (SD). Differences between results for respective oils marked with the same letter in the same row are statistically in significant (*p* < 0.05). The abbreviation “nd” indicates fatty acids that were not detected.

**Table 2:** Determined values of specific absorbance coefficients, *K*_232_ and *K*_268_, during the storage test.

Storage Time [Day]	MTSO	EPSO	FSO	CSSO	BCSO	PSO
*K*_232_						
0	6.02 ± 0.31 ^e^	7.60 ± 0.40 ^e^	1.80 ± 0.10 ^f^	1.60 ± 0.10 ^f^	5.60 ± 0.30 ^c^	7.50 ± 0.10 ^d^
3	12.22 ± 1.24 ^d^	15.10 ± 0.90 ^d^	3.10 ± 0.10 ^e^	3.80 ± 0.10 ^e^	5.80 ± 0.40 ^c^	6.80 ± 0.30 ^e^
6	14.65 ± 0.82 ^d^	17.10 ± 1.20 ^d^	3.10 ± 0.20 ^e^	6.10 ± 0.30 ^d^	5.70 ± 0.20 ^c^	6.80 ± 0.40 ^e^
10	18.93 ± 1.26 ^c^	29.90 ± 0.60 c	7.20 ± 0.50 ^d^	9.30 ± 0.80 ^c^	5.40 ± 0.40 ^c,d^	9.10 ± 0.60 ^c^
14	32.40 ± 2.25 ^a^	57.20 ± 2.20 ^b^	13.70 ± 1.20 ^c^	12.40 ± 1.00 ^b^	5.90 ± 0.20 ^c^	9.10 ± 0.80 ^c^
18	29.62 ± 1.31 ^b^	109.70 ± 6.30 ^a^	26.70 ± 2.10 ^b^	24.50 ± 1.80 ^a^	6.50 ± 0.10 ^b^	12.60 ± 0.70 ^b^
21	35.21 ± 1.81 ^a^		57.90 ± 1.80 ^a^	22.70 ± 0.80 ^a^	8.00 ± 0.80 ^a^	16.00 ± 1.30 ^a^
*K*_268_						
0	0.31 ± 0.01 ^f^	0.07 ± 0.00 ^e^	0.14 ± 0.01 ^e^	0.03 ± 0.00 ^f^	1.34 ± 0.11 ^a,b^	2.70 ± 0.21 ^a^
3	0.64 ± 0.04 ^e^	0.39 ± 0.03 ^d^	0.16 ± 0.01 ^e^	0.10 ± 0.01 ^e^	1.42 ± 0.04 ^a^	2.20 ± 0.23 ^a,b^
6	0.60 ± 0.06 ^e^	0.47 ± 0.03 ^d^	0.12 ± 0.01 ^e^	0.28 ± 0.03 ^d^	1.30 ± 0.06 ^b^	2.40 ± 0.16 ^a^
10	0.97 ± 0.05 ^d^	1.23 ± 0.52 ^c^	0.28 ± 0.01 ^d^	0.64 ± 0.01 ^c^	1.10 ± 0.05 ^c^	2.20 ± 0.11 ^a,b^
14	1.57 ± 0.03 ^c^	2.59 ± 0.32 ^b^	0.47 ± 0.03 ^c^	1.24 ± 0.03 ^b^	1.12 ± 0.03 ^c^	2.20 ± 0.20 ^a,b^
18	2.41 ± 0.15 ^b^	10.46 ± 0.10 ^a^	1.18 ± 0.11 ^b^	2.72 ± 0.03 ^a^	1.02 ± 0.10 ^c,d^	2.30 ± 0.10 ^a^
21	3.71 ± 0.28 ^a^	-	2.65 ± 0.21 ^a^	2.69 ± 0.07 ^a^	1.04 ± 0.08 ^c,d^	2.50 ± 0.11 ^a^

Explanatory notes: The data in the table are presented as the mean ± standard deviation (SD). Differences between results for respective oils marked with the same letter in the same row are statistically insignificant (*p* < 0.05).

**Table 3:** The parameters designated from the isotherm profile.

Parameter	MTSO	EPSO	FSO	CSSO	BCSO	PSO
A_EXP_ [cm^2^]	114.4. ± 2.4 ^e^	147.6 ± 4.1 ^c^	147.2 ± 2.9 ^c^	135.6 ± 1.7 ^d^	182.4 ± 5.2 ^a^	174.7 ± 4.6 ^b^
A_C_ [cm^2^]	45.6 ± 0.3 ^c^	45.6 ± 0.6 ^c^	45.6 ± 0.2 ^c^	45.6 ± 0.1 ^c^	50.8 ± 0.9 ^b^	52.9 ± 1.0 ^a^
π_c_ [mN/m]	11.4 ± 0.1 ^d^	10.4 ± 0.1 ^e^	10.7 ± 0.2 ^e^	14.3 ± 0.1 ^c^	16.4 ± 0.3 ^b^	25.3 ± 0.6 ^a^

Explanatory notes: The parameters designated from the isotherm profile: A_EXP_—the extrapolated average surface area available to the molecule; A_C_—the average surface area available to the molecule at the collapse point; and π_c_—the surface pressure at the collapse point. The data in the table are presented as the mean ± standard deviation (SD). Differences between results for respective oils marked with the same letter in the same row are statistically in significant (*p* < 0.05).

**Table 4:** Determined values of the modulus of compression (C_S_^−1^) and viscoelastic properties (ε).

Parameter	MTSO	EPSO	FSO	CSSO	BCSO	PSO
C_S_^−1^ _MAX_ [mN/m]	86.3 ± 1.8 ^c^	72.0 ± 1.4 ^d^	84.0 ± 2.0 ^c^	104.7 ± 2.8 ^b^	136.4 ± 5.2 ^a^	138.5 ± 4.6 ^a^
ε_MAX_ [mN/m]	74.2 ± 0.9 ^d^	68.9 ± 0.7 ^d^	70.3 ± 1.1 ^d^	94.8 ± 2.1 ^c^	112.6 ± 5.9 ^b^	122.3 ± 4.8 ^a^

Explanatory notes: The data in the table are presented as the mean ± standard deviation (SD). Differences between results for respective oils marked with the same letter in the same row are statistically in significant (*p* < 0.05).

## Data Availability

The original contributions presented in the study are included in the article; further inquiries can be directed to the corresponding authors.
