# Correction: Kamińska et al. Behavior at Air/Water Interface and Oxidative Stability of Vegetable Oils Analyzed Through Langmuir Monolayer Technique. *Molecules* 2025, *30*, 170

**DOI:** 10.3390/molecules31172995

**Published:** 2026-08-27

**Authors:** Wiktoria Kamińska, Katarzyna Rzyska-Szczupak, Anna Przybylska-Balcerek, Kinga Stuper-Szablewska, Anna Dembska, Grażyna Neunert

**Affiliations:** 1 Department of Physics and Biophysics, Faculty of Food Science and Nutrition, Poznan University of Life Sciences, Wojska Polskiego 38/42, 60-637 Poznan, Poland; 2 Department of Chemistry, Faculty of Forestry and Wood Technology, Poznan University of Life Sciences, 60-628 Poznan, Poland; katarzyna.rzyska@up.poznan.pl (K.R.-S.); anna.przybylska@up.poznan.pl (A.P.-B.); kinga.stuper@up.poznan.pl (K.S.-S.); 3 Department of Bioanalytical Chemistry, Faculty of Chemistry, Adam Mickiewicz University, Uniwersytetu Poznanskiego 8, 61-614 Poznan, Poland; aniojka@amu.edu.pl

We were notified that there were errors in the original publication [1].

## 1. Errors in Tables and Figures

During manuscript preparation, the percentages of several fatty acids in Table 1 for FSO and CSSO were accidentally swapped (C14:0 with C16:0 and C18:2 with C18:3 for FSO and CSSO, respectively). In addition, C20:2 was written instead of C20:1, and an incorrect total MUFA value and an incorrect SD value for C18:2 were reported for FSO. Moreover, the position of the letters denoting statistically significant differences for PSO in rows C18:0, C18:1 and C18:2 was incorrect. The changes introduced in Table 1 required a correction of the significance of differences between the results for individual oils. 

During data editing for Table 3, the π_c_ values for MTSO, FSO, and EPSO were accidentally interchanged and incorrect letters denoting a statistically significant difference for these oils were entered. This error requires correction of the data in Table 3, as well as the replacement of the corresponding curves in Figure 3d. In addition, an incorrect A_C_ value, together with a letter denoting a statistically significant difference, was entered for EPSO, and an incorrect π_c_ value was entered for BCSO in Table 3. 

During the same editing process, the C_S_^−1^ and ε values for two oils (EPSO and FSO) were also accidentally interchanged. This error requires rearrangement of the data in Table 4, as well as the replacement of the corresponding curves for FSO and EPSO in Figure 4a,b. Furthermore, Figure 4b contained an incorrect relationship for PSO, which has also been corrected.

In Figure 1a, the curve for PSO after three days of oxidation was mistakenly included instead of the curve for the fresh oil. This error requires replacement of the incorrect curve in Figure 1.

The corrected Table 1, Table 3 and Table 4 and Figure 1, Figure 3 and Figure 4 appear below.

**Table 1:** The fatty acid profile (FAME) in investigated oils.

Fatty Acid [%]	MTSO	EPSO	FSO	CSSO	BCSO	PSO
C14:0	1.76 ± 0.13 ^a^	nd	nd	nd	0.16 ± 0.01 ^c^	0.28 ± 0.01 ^b^
C16:0	nd	8.74 ± 0.05 ^d^	11.59 ± 0.17 ^b^	8.46 ± 0.05 ^d^	11.20 ± 0.10 ^c^	12.60 ± 0.02 ^a^
C16:1	0.55 ± 0.06 ^c^	nd	nd	2.70 ± 0.10 ^a^	1.20 ± 0.15 ^b^	0.55 ± 0.01 ^c^
C18:0	4.43 ± 0.04 ^c^	3.31 ± 0.10 ^d^	5.73 ± 0.05 ^b^	0.36 ± 0.04 ^e^	0.33 ± 0.04 ^e^	8.30 ± 0.02 ^a^
C18:1	25.87 ± 0.45 ^a^	17.32 ± 0.25 ^d^	20.73 ± 0.21 ^c^	20.52 ± 0.30 ^c^	26.42 ± 0.87 ^a^	24.05 ± 0.31 ^b^
C18:2	47.70 ± 0.42 ^d^	70.30 ± 0.20 ^a^	14.28 ± 0.18 ^e^	6.75 ± 0.05 ^f^	55.5 ± 0.36 ^b^	52.41 ± 0.17 ^b,c^
C18:3, n-3	14.23 ± 0.13 ^c^	0.24 ± 0.04 ^f^	46.73 ± 0.18 ^b^	52.70 ± 0.20 ^a^	1.21 ± 0.10 ^d^	0.83 ± 0.02 ^e^
C18:3, n-6	0.16 ± 0.01 ^a^	nd	0.15 ± 0.01 ^a^	nd	nd	nd
C20:0	3.48 ± 0.04 ^a^	nd	0.55 ± 0.01 ^c^	3.49 ± 0.04 ^a^	1.23 ± 0.15 ^b^	nd
C20:1	0.18 ± 0.01 ^b^	nd	0.17 ± 0.01 ^b^	0.18 ± 0.01 ^b^	0.33 ± 0.05 ^a^	nd
SFA	9.67 ± 0.02 ^e^	12.05 ± 0.04 ^d^	17.87 ± 0.02 ^b^	12.31 ± 0.01 ^d^	12.92 ± 0.01 ^c^	21.18 ± 0.20 ^a^
MUFA	26.42 ± 0.32 ^a^	17.32 ± 0.23 ^d^	20.73 ± 0.05 ^c^	23.22 ± 0.31 ^b^	27.62 ± 0.42 ^a^	24.61 ± 0.21 ^b^
PUFA	62.09 ± 0.71 ^b^	70.54 ± 0.32 ^a^	61.16 ± 0.56 ^b^	59.45 ± 0.81 ^b,c^	56.71 ± 0.42 ^c,d^	53.24 ± 0.32 ^d^

Explanatory notes: The data in the table are presented for investigated oils: milk thistle oil (MTSO), evening primrose seed oil (EPSO), flaxseed oil (FSO), camelina sativa seed oil (CSSO), black cumin seed oil (BCSO), and pumpkin seed oil (PSO). The data in the table are presented as the mean ± standard deviation (SD). Differences between results for respective oils marked with the same letter in the same row are statistically in significant (*p* < 0.05). The abbreviation “nd” indicates fatty acids that were not detected.

**Table 3:** The parameters designated from the isotherm profile.

Parameter	MTSO	EPSO	FSO	CSSO	BCSO	PSO
A_EXP_ [cm^2^]	114.4. ± 2.4 ^e^	147.6 ± 4.1 ^c^	147.2 ± 2.9 ^c^	135.6 ± 1.7 ^d^	182.4 ± 5.2 ^a^	174.7 ± 4.6 ^b^
A_C_ [cm^2^]	45.6 ± 0.3 ^c^	45.6 ± 0.6 ^c^	45.6 ± 0.2 ^c^	45.6 ± 0.1 ^c^	50.8 ± 0.9 ^b^	52.9 ± 1.0 ^a^
π_c_ [mN/m]	11.4 ± 0.1 ^d^	10.4 ± 0.1 ^e^	10.7 ± 0.2 ^e^	14.3 ± 0.1 ^c^	16.4 ± 0.3 ^b^	25.3 ± 0.6 ^a^

Explanatory notes: The parameters designated from the isotherm profile: A_EXP_—the extrapolated average surface area available to the molecule; A_C_—the average surface area available to the molecule at the collapse point; and π_c_—the surface pressure at the collapse point. The data in the table are presented as the mean ± standard deviation (SD). Differences between results for respective oils marked with the same letter in the same row are statistically in significant (*p* < 0.05).

**Table 4:** Determined values of the modulus of compression (C_S_^−1^) and viscoelastic properties (ε).

Parameter	MTSO	EPSO	FSO	CSSO	BCSO	PSO
C_S_^−1^ _MAX_ [mN/m]	86.3 ± 1.8 ^c^	72.0 ± 1.4 ^d^	84.0 ± 2.0 ^c^	104.7 ± 2.8 ^b^	136.4 ± 5.2 ^a^	138.5 ± 4.6 ^a^
ε_MAX_ [mN/m]	74.2 ± 0.9 ^d^	68.9 ± 0.7 ^d^	70.3 ± 1.1 ^d^	94.8 ± 2.1 ^c^	112.6 ± 5.9 ^b^	122.3 ± 4.8 ^a^

Explanatory notes: The data in the table are presented as the mean ± standard deviation (SD). Differences between results for respective oils marked with the same letter in the same row are statistically in significant (*p* < 0.05).

**Figure 1:**
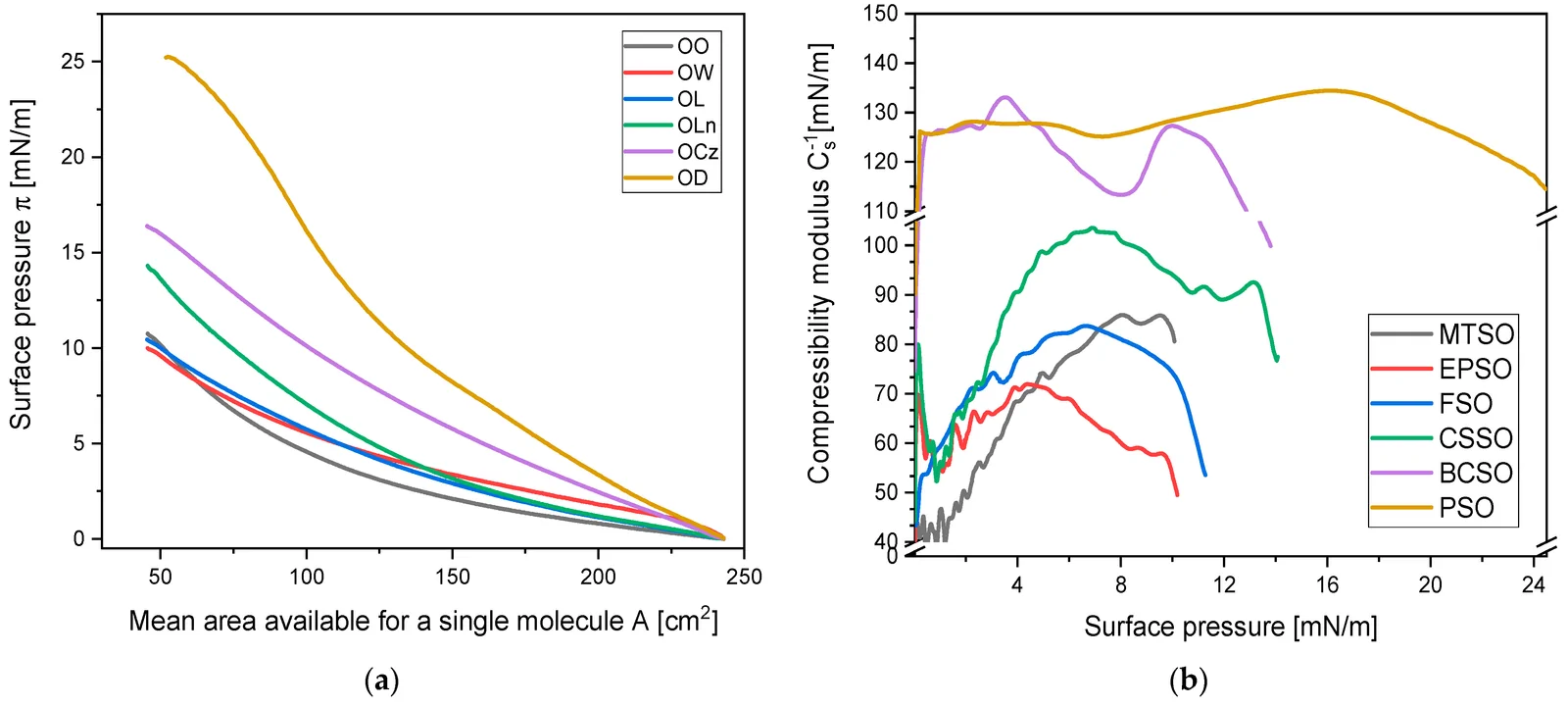
Compression π-A isotherms of Langmuir monolayers (**a**) and compression modulus–surface pressure dependences (**b**) for the tested oils.

**Figure 3:**
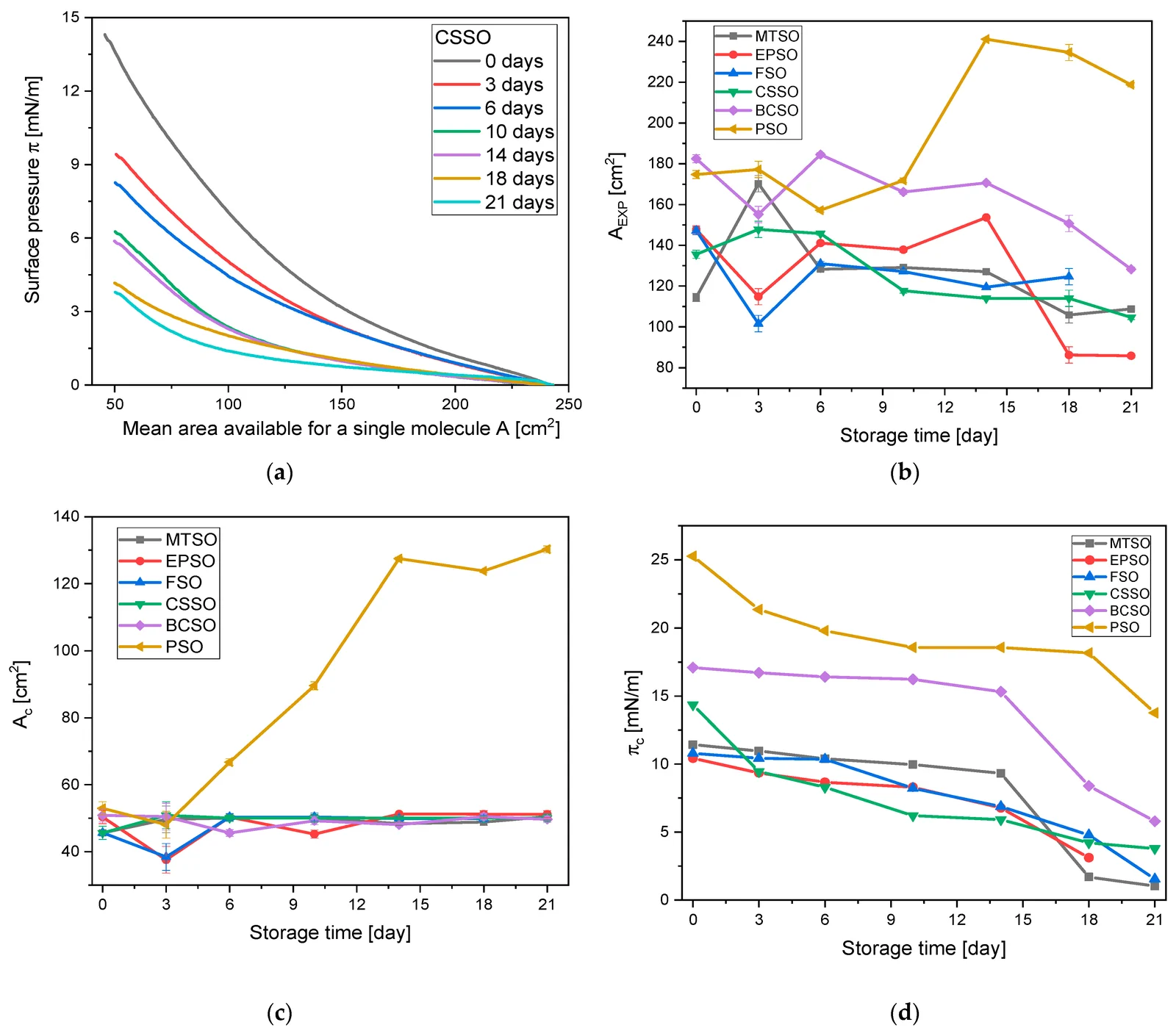
Compression isotherms of Langmuir monolayers for CSSO on different days of storage (**a**), dependences of the extrapolated mean molecular area (A_EXT_), (**b**) and the mean molecular area at the collapse point (A_C_) on the tested oil (**c**), as well as the dependence of the surface pressure at the collapse point (π_c_) on the tested oil (**d**).

**Figure 4:**
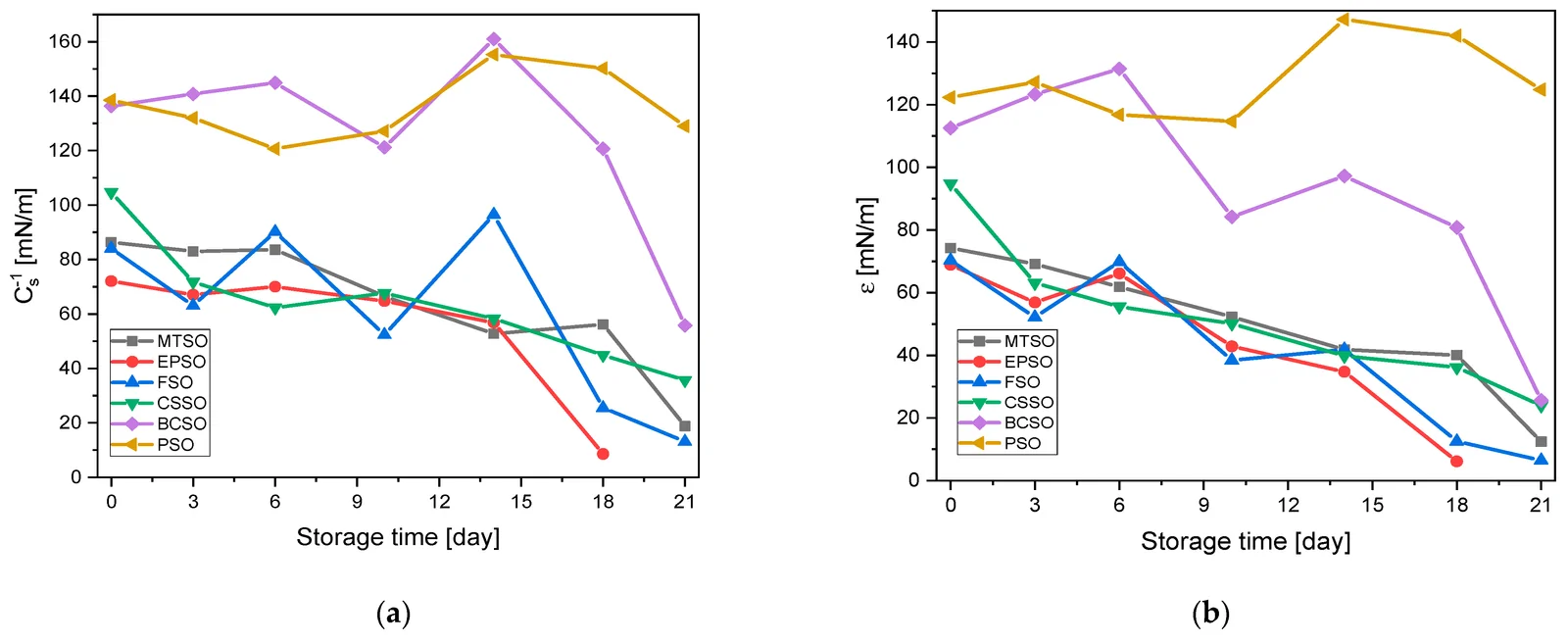
Dependence of the compressibility modulus C_S_^−1^ (**a**) and the viscoelastic modulus ε (**b**) on time for the tested oil.

## 2. Text Corrections

Following the correction of the data in Table 1, one sentence in Section 2.1 describing the fatty acid profile of CSSO has been revised by replacing the term “linoleic acid” with “α-linolenic acid.” The corrected text appears below: “CSSO was characterized by 59.45% PUFA, primarily α-linolenic acid (52.70%), and 20.52% MUFA, mainly oleic acid, with an SFA content of 12.31%, consistent with values reported in previous studies [29].”

Because the data for FSO and EPSO were interchanged, the abbreviations for linseed oil (FSO) and evening primrose oil (EPSO) have been corrected in several sentences in Sections 2.3.2 and 2.4.2. The corrected sentences are provided below: Section 2.3.2: “MTSO and FSO showed ε values of approximately 74.2 mN/m and 70.3 mN/m, respectively, indicating that they formed less elastic monolayers while remaining within the characteristic range of the second zone. Finally, EPSO exhibited the lowest ε value (68.9 mN/m), indicating lower rigidity than the other oils but still sufficient elasticity to form a stable monolayer.”

Section 2.4.2: “The most pronounced decrease was observed for FSO and EPSO. After 21 days, the CS^−1^ values decreased to 13.1 mN/m and 8.6 mN/m, respectively, corresponding to the LE and L phases.”

Section 2.4.2: “The lowest ε values were observed for FSO and EPSO. After 21 days, they reached 6.5 mN/m and 6.2 mN/m, respectively, indicating a substantial loss of elasticity and monolayer stability.”

In the last sentence of Section 2.3.3, the abbreviations for FSO and BCSO were accidentally interchanged and have now been corrected. The corrected sentence appears below: “For PSO and FSO, which contained higher levels of SFA, the ε correlations were stronger than those observed for BCSO and CSSO.”

The authors state that the scientific conclusions are unaffected. This correction was approved by the Academic Editor. The original publication has also been updated.
